# Asthma and obesity increase inflammatory markers in children

**DOI:** 10.3389/falgy.2024.1536168

**Published:** 2025-01-20

**Authors:** Harshita Shailesh, Safa Noor, Lena Hayati, Antonisamy Belavendra, Nicholas Van Panhuys, Abdul Badi Abou-Samra, Stefan Worgall, Ibrahim Janahi

**Affiliations:** ^1^ Department of Pediatric Medicine, Division of Pulmonology, Sidra Medicine, Doha, Qatar; ^2^ Laboratory of Immunoregulation, Sidra Medicine, Doha, Qatar; ^3^ Academic Health System, Hamad Medical Corporation, Qatar Metabolic Institute, Doha, Qatar; ^4^ Department of Pediatrics, Weill Cornell Medical College, New York, NY, United States; ^5^ Department of Pediatrics, Weill Cornel Medicine-Qatar (WCM-Q), Doha, Qatar

**Keywords:** asthma, obesity, inflammation, cytokines, pediatric asthma

## Abstract

**Background:**

Asthma and obesity are both characterized by inflammation. However, the combined impact of these conditions on inflammatory mechanisms in children has not been studied extensively. To address this gap, we investigated the interaction effects of asthma and obesity on inflammation in children.

**Methods:**

The multiplex and singleplex assays were used to measure the levels of circulating cytokines, including IL-2, IL-5, IL-10, IL-13, IL-17A, IL-22, IL-33, IFN-γ, TNF-α, and the adipokine leptin, in plasma. The study included 97 children with normal weight and asthma (NW-A), 100 children with overweight/obesity and asthma (OO-A), 100 with overweight/obesity and no asthma (OO), and 67 normal weight children and no asthma (NW). The independent effects of asthma, obesity, and their interaction effect on these inflammatory markers were assessed using multiple regression analysis.

**Results:**

Asthma was associated with the increased expression of pro-inflammatory cytokines, including IL-2, IL-5, IL-13, IL-17A, IL-22, IL-33, and TNF-α, and reduced levels of anti-inflammatory cytokine, IL-10 and adipokine, leptin in the circulation. Overweight/obesity was also linked to increased plasma levels of IL-5, IL-17A, IL-22, IL-33, TNF-α, and leptin and decreased levels of IL-10. In addition, obesity and asthma showed a significant interaction effect on the plasma levels of IL-5, IL-10, IL-17A, IL-33, TNF-α, and leptin. However, the interaction did not result in a synergistic or additive impact on cytokines, indicating a moderating effect of obesity on inflammation in pediatric asthma.

**Conclusion:**

Both asthma and overweight/obesity were independently associated with increased expression of pro-inflammatory cytokines and decreased expression of anti-inflammatory cytokine in children. While the concurrent presence of asthma and obesity altered the inflammatory profile, it did not synergistically amplify the inflammation. These findings challenge the previous view that obesity enhances inflammation in individuals with asthma and highlight the importance of considering both conditions while treating obesity-associated asthma in children. Future studies are necessary to further explore the mechanisms that link obesity and asthma in the pediatric population.

## 1 Introduction

Asthma is the most heterogeneous lung disease, characterized by coughing, shortness of breath, wheezing, and chest tightness, driven by underlying chronic inflammation of the airway (1). Exposure to allergens and environmental triggers in the airway initiates a cascade of events, resulting in the secretion of multiple cytokines, including interleukin (IL)-4, IL-5, and IL-13, and chemokines such as IL-25, IL-33, and TSLP by lung-resident immune cells and structural cells of the airway (2, 3). These cytokines and chemokines play pivotal roles in orchestrating airway inflammation by recruiting and activating members of the innate immune system, including basophils, eosinophils, macrophages, and neutrophils, enhancing mucus secretion, and inducing hyperresponsiveness and remodeling of the airway (3, 4).

Recent advances in understanding asthma pathogenesis have made it clear that various cytokines, in addition to the atopy-associated Th2 cytokines (IL-4, IL-5, and IL-13), play crucial roles in the induction and regulation of airway inflammation. Dysregulated expression of the anti-inflammatory cytokines (IL-10), Th1-derived cytokine [interferon-gamma (IFN-γ)], and the Th17-cell cytokines (IL-17A, IL-17F, and IL-22) both in the airway and circulation, are often reported in asthmatic patients (5–7). Recently, multiple studies have focused on the association between Th2 and non-Th2 cytokines and the clinical features of asthma to better understand their pathogenic roles and develop targeted therapies (8).

The obesity epidemic is a risk factor for increased asthma incidence in children, as demonstrated by various case-control and cross-sectional studies (9, 10). Whilst the complex interplay between obesity and asthma is not yet completely understood, studies in pre-clinical models have revealed that obesity-associated chronic low-grade inflammation and adipokines such as leptin promote inflammation and remodeling of the lungs (11, 12). High-fat-fed obese mice show increased neutrophilic inflammation, and elevated expression of cytokines including TNF-α, IL-1β, TGF-β and IL-17 in the lung after allergen exposure (13). However, studies in adults with asthma and obesity have reported varying results, with some indicating that obesity promotes neutrophilic inflammation in the airway (14, 15). In contrast, others have found that obesity does not influence airway inflammation (16, 17). On the other hand, the impact of obesity on inflammation in the pediatric population with asthma has not been studied extensively. Clinically, asthmatic children with obesity show an increased need for inhaled and oral steroids, indicating a state of enhanced inflammation in these children (18, 19). Given the rising global prevalence of obesity in children in recent years, understanding how obesity interacts with asthma to modify inflammation is crucial for developing effective management strategies. Here, we hypothesized that children with both asthma and overweight/obesity would exhibit a synergistically elevated pro-inflammatory cytokine profile compared to those with only one condition. We tested this hypothesis by comparing plasma cytokines, including IL-2, IL-5, IL-10, IL-13, IL-17A, IL-22, IL-33, IFN-γ, and TNF-α and the adipokine, leptin in normal weight and overweight/obese children, both with and without asthma in a cross-sectional study. Additionally, we also explored whether the interaction between obesity and asthma reduces lung function in children.

## 2 Methods

### 2.1 Study population

**Figure 1:**
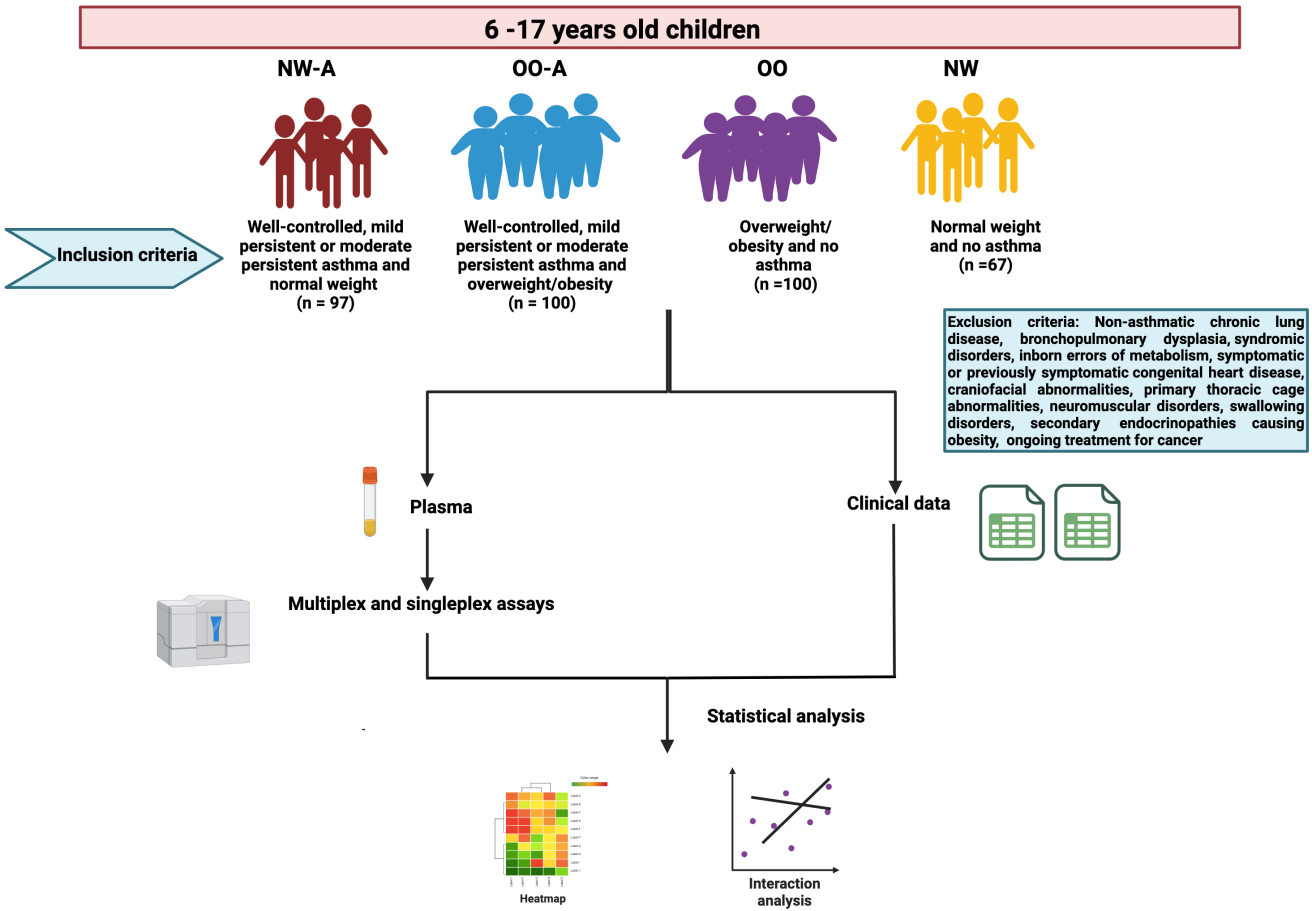
Flowchart of the study.

This study is part of the Sphingolipids in Obesity and Asthma in Pediatrics (SOAP), a large, multi-purpose cross-sectional study designed to characterize the physiological, genetic, epigenetic, metabolomic, and lipidomic factors that influence asthma and obesity in children residing in Qatar. The study was approved by the Institutional Review Board (IRB: 1500770) at Sidra Medicine, Doha, Qatar. Recruitment details of the SOAP study have been described in detail elsewhere (20). The flowchart of the current study along with inclusion and exclusion criteria are summarized in the Figure 1. Briefly, children of 6–17 years of age, attending outpatient clinics at Sidra Medicine, Qatar, were recruited and classified into the following groups based on their asthma status and body weight: (1) normal weight and asthma (well-controlled, mild persistent or moderate persistent) (NW-A) (*n* = 97), (2) overweight/obesity and asthma (well-controlled, mild persistent or moderate persistent) (OO-A) (*n* = 100), (3) overweight/obesity and no asthma (OO) (*n* = 100), and (4) normal weight and no asthma (NW) (*n* = 67). The study excluded the children with non-asthmatic chronic lung disease, bronchopulmonary dysplasia, syndromic disorders, inborn errors of metabolism, symptomatic or previously symptomatic congenital heart disease, craniofacial abnormalities, primary thoracic cage abnormalities, neuromuscular disorders, swallowing disorders, secondary endocrinopathies causing obesity, and ongoing treatment for cancer.

Asthma criteria were based on a physician's diagnosis and a documented history of asthma symptoms, in accordance with the protocols of the Global Initiative of Asthma (GINA) guidelines (21). Additionally, a confirmed asthma diagnosis at least six months prior to the recruitment, irrespective of lung function test results and ongoing asthma treatment was necessary. The children with mild persistent or moderate persistent asthma in a well-controlled status according to physician's diagnosis and GINA guidelines were included in the study. Overweight and obesity criteria were based on a body mass index (BMI) above the 85th and 95th percentile for age and sex, respectively. A summary of the demographic and clinical characteristics of the groups is presented in Table 1.

**Table 1:** Basic characteristics of the study participants (*n* = 364).

Variable	Norma weight and asthma (NW-A) (*n* = 97)	Overweight/obesity and asthma (OO-A) (*n* = 100)	Over-weight obesity and no asthma (OO) (*n* = 100)	Normal weight and no asthma (NW) (*n* = 67)	*p* value^c^
Age (years)^a^	10.87 (3.1)	12.04 (3.4)	12.40 (2.9)	10.67 (3.1)	<0.001
Gender, *n* (%)					
Male	72 (74.2)	73 (73.0)	45 (45.0)	36 (53.7)	<0.001
Female	25 (25.8)	27 (27.0)	55 (55.0)	31 (46.3)	
BMI (kg/m^2^)^b^	16.7 (15.1, 18.6)	26.3 (22.5, 30.2)	31.4 (24.8, 38.3)	16.2 (14.9, 18.0)	<0.001
BMI Z-score^b^	−0.14 (−0.78, 0.50)	1.93 (1.52, 2.31)	2.18 (1.83, 2.62)	−0.31 (−.97, 0.22)	<0.001
BMI-percentile^b^	44.5 (21.9, 69.1)	97.3 (93.6, 98.9)	98.5 (96.7, 99.6)	37.9 (16.5, 58.9)	<0.001
Eczema, n (%)	35 (36.1)	23 (23.0)	9 (9.0)	0 (0.0)	<0.001
Allergies, *n* (%)	35 (36.1)	31 (31.0)	7 (7.0)	1 (1.5)	<0.001
Rhinitis, *n* (%)	45 (46.4)	51 (51.0)	3 (3.0)	2 (3.0)	<0.001
Eosinophils (×10^9 ^/L)^b^	0.35 (0.2, 0.6)	0.3 (0.2, 0.5)	0.2 (0.1, 0.3)	0.2 (0.1, 0.3)	<0.001
Eosinophilia status (≥0.3 × 10^9 ^/L), *n* (%)	61 (63.5)	54 (56.3)	29 (29.9)	17 (25.8)	<0.001
Neutrophils (×10^9 ^/L)^b^	2.5 (1.9, 3.5)	3.1 (2.5, 4.3)	3.7 (2.9, 4.6)	2.5 (1.6, 3.4)	<0.001
FeNO ≥ 20 ppb, *n* (%)	65 (73.9)	65 (67.7)	37 (42.5)	26 (44.1)	<0.001
Asthma medication					
Montelukast, *n* (%)	27 (27.8)	33 (33.0)	–	–	0.43
Inhaled steroids, *n* (%)	31 (32.0)	31 (31.0)	–	–	0.89
Inhaled steroids and LABA, *n* (%)	17 (17.5)	20 (20.0)	–	–	0.66
Systemic glucocorticoids, *n* (%)	5 (5.2)	5 (5.0)	–	–	0.961

FeNO, fractional exhaled nitric oxide; LABA, long-acting beta agonists.

^a^ Mean (SD).

^b^ Median (interquartile range); BMI Z-score for age and gender.

^c^ One-way ANOVA or Kruskal–Wallis test or chi-square test.

### 2.2 Pulmonary function tests

Pulmonary function tests, including spirometry, body plethysmography, lung clearance index, and fractional nitric oxide measurements (FeNO) were carried out for the participants, as explained elsewhere (20).

### 2.3 Cytokine assay

From each participant, 3 ml blood was collected in an EDTA tube, which was then centrifuged at 1,500 × g for 15 min at 4°C. The plasma obtained after centrifugation was stored at −80°C. For cytokine analysis, frozen plasma samples were thawed and vortexed vigorously for 2 min, followed by centrifugation at 10,000 RCF for 15 min at room temperature. Custom designed MILLIPLEX MAP Human TH17 Magnetic Bead Panel—Immunology Multiplex Assay kit (Merck Millipore, USA, Catalog number # HTH17MAG-14K), a magnetic bead based assay, was used to measure levels of cytokines (IL-2, IL-5, IL-10, IL13, IL-17A, IL-22, IL-33, IFN-γ, and TNF-α) in the plasma samples according to the manufacturer's instructions. The panel was designed by the manufacturer to have minimal cross-reactivity between the antibodies and analytes, ensuring high specificity in the cytokine measurement. Neat plasma samples were used for cytokine measurement as per the manufacturer's instructions. The minimum detectable concentration for cytokines were IL-2 (9 pg/ml), IL-5 (1.5 pg/ml), IL-10(0.5 pg/ml), IL-13 (3.5 pg/ml), IL-17A (2.8 pg/ml), IL-22 (0.32 ng/ml), IL-33 (10 pg/ml) IFN-γ (2.4 pg/ml), and TNF-α (1.7 pg/ml). Leptin was measured using Bio-Plex Pro Human Diabetes single plex custom designed assay (Bio-Rad Laboratories Ltd, Hertfordshire, U.K, Catalog number # #171A7001M). Plasma samples were diluted 4 times with assay buffer for leptin measurement as per the manufacturer's instructions. The minimum detectable concentration for leptin was 3 pg/ml. All the standards and samples were run in duplicates and the average values were used in subsequent analysis. The pre-mixed quality control samples provided by the manufacturer was used to validate the assay performance. Five-parameter logistic regression algorithms built into the Bioplex manager six software was used to assess levels of cytokines and leptin levels in reference to standards. Analysis was conducted using a Bioplex-200 instrument according to the manufacturer's instructions (BIO-RAD, Hertfordshire, UK). Among the 364 participants, we were unable to detect cytokines in 2 subjects (each from OO-A, and OO group) due to technical issues.

### 2.4 Statistical analysis

Results are presented as summary statistics, using mean and standard deviation (SD) for quantitative variables that were normally distributed and median with interquartile range (IQR) for skewed variables. Categorical data are presented using counts and percentages. Comparisons between groups were made using one-way analysis of variance (ANOVA) or the Kruskal–Wallis test for quantitative variables and the chi-square test for categorical variables. Sample size was calculated based on pilot data examining serum ceramide/dihydroceramide ratio in children. We needed 90 subjects per group to detect a difference in the mean ratios of 0.25 (unitless ratio) between children with non-atopic asthma (mean 1.47) and those with no atopy and no asthma (mean 1.22), with a standard deviation of 0.6 at 80% power and a 5% level of significance.

Pulmonary function, body plethysmography tests, and cytokine concentrations were used to assess the effects of asthma, obesity, and their interaction using multiple regression analysis. Geometrical mean of the cytokines was used to calculate log fold change values. One-way analysis of variance using Bonfrerroni multiple comparison method was used for comparison of each study group with control. A *p* value <0.05 was considered significant. All the analyses were performed using STATA/SE 18.0 (StataCorp, College Station, Texas 77845, USA).

## 3 Results

### 3.1 Demographics and clinical characteristics

The mean age of children in the OO-A group was higher than that of the NW-A group (Table 1). Similarly, children in the OO were older than those in the NW category. There was no significant difference in sex distribution between the OO-A and NW-A groups. However, the number of females was higher in the non-asthma groups (OO and NW) than in the asthma groups (NW-A and OO-A). As expected, BMI Z-score and BMI-percentile were higher in children with overweight/obesity (OO and OO-A) compared to those with normal weight children (NW and NW-A), irrespective of asthma status (*p* < 0.001) (Table 1). Children with asthma had significantly more histories of atopy, including eczema, allergies, and rhinitis (*p* < 0.001), compared to children without asthma. However, the incidence of atopic comorbidities did not differ significantly between the OO-A and the NW-A group. Total blood eosinophils and incidence of eosinophilia (≥0.30 × 10^9 ^/L) were higher in children with asthma than non-asthma (*p* < 0.001), irrespective of their weight status. However, these parameters did not differ significantly between the OO-A group and the NW-A group. Notably, children with overweight/obesity had a higher blood neutrophil count than their normal weight counterparts, irrespective of asthma status (*p* < 0.001). The proportion of participants with FeNO ≥ 20 ppb was significantly higher in both the asthma groups, compared to non-asthma groups. There was no significant difference in asthma medication intake between the NW-A group and the OO-A group (Table 1).

### 3.2 Inflammatory markers

**Figure 2:**
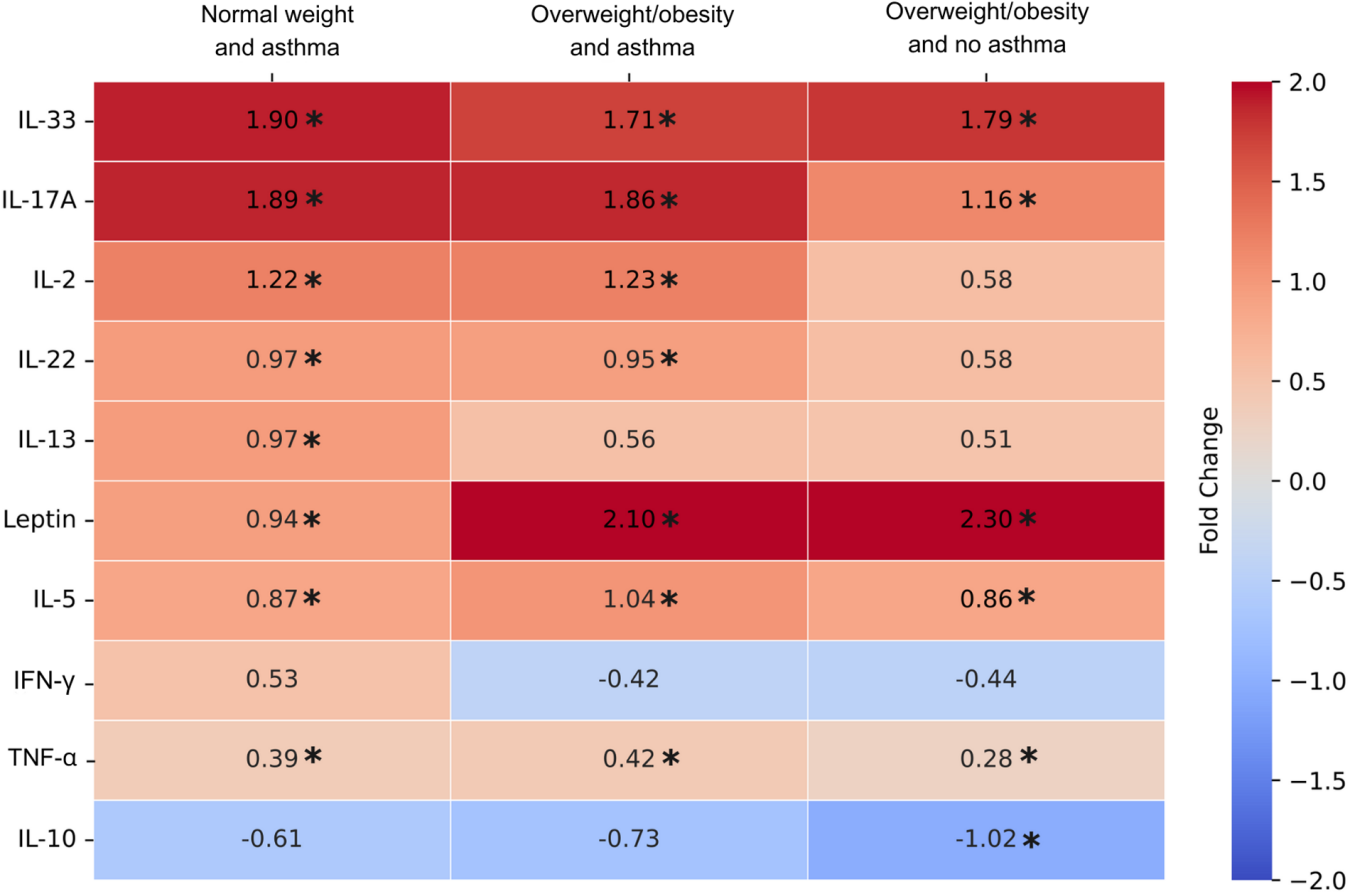
Log fold changes of plasma cytokine levels in normal weight and asthma (NW-A), overweight/obesity and asthma (OO-A) and overweight/obesity and no asthma (OO) groups as compared to normal weight and no asthma (NW) group. Log fold changes were obtained for NW-A, OO-A and OO compared to the NW group using one-way analysis of variance using Bonfrerroni multiple comparison method and included in the heatmap. The comparisons with *p* values < 0.05 are indicated with asterisk.

The results of plasma cytokine analysis indicated that pro-inflammatory cytokines including IL-33, IL-17A, leptin, IL-5, and TNF-α were consistently increased in NW-A, OO-A, and OO groups compared to the NW control group (Figure 2). Next, the levels of IL-2 and IL-22 showed a significant increase only in asthma groups (NW-A and OO-A) compared to the NW group. The IL-13 level was significantly upregulated only in the NW-A group compared to the NW control group. Although, IL-10 was consistently decreased across all the study groups compared to the control group, only the OO group showed a statistically significant reduction. The levels of IFN-γ did not show significant variation across the groups (Figure 2).

**Figure 3:**
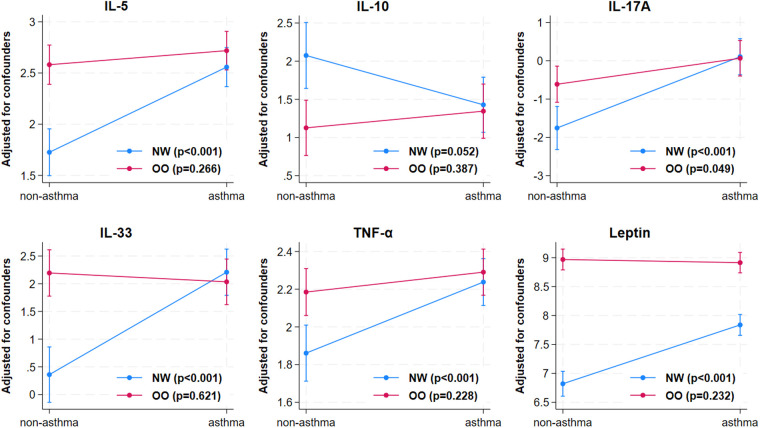
Interaction effect of obesity and asthma on cytokines. The cytokines including IL-5, IL-10, IL-17A, IL-33, TNF-α, and leptin were significantly affected by the interaction of asthma and obesity. NW, normal weight; OO, overweight/obesity; IL, interleukin; TNF-α, tumor necrosis factor-alpha.

Next, to understand the independent impacts of asthma and obesity and their interaction effect on plasma inflammatory markers, a regression analysis was carried out after correcting for cofounders including age, gender, and medication use. Children with asthma had higher levels of IL-2 (*p* = 0.005), IL-5 (*p* < 0.001), IL-13 (*p* = 0.021), IL-17A (*p* < 0.001), IL-22 (*p* < 0.001), IL-33 (*p* < 0.001), TNF-α (*p* < 0.001), and lower levels of IL-10 (*p* = 0.046) and leptin (*p* < 0.001) compared to those without asthma (Table 2). Children with overweight/obesity had higher IL-5 (*p* < 0.001), IL-17A (*p* = 0.002), IL-22 (*p* = 0.030), IL-33 (*p* < 0.001), TNF-α (*p* = 0.001), and leptin (*p* < 0.001), and lower IL-10 (*p* = 0.002) levels compared to normal weight children, indicating that overweight/obesity affects these cytokines regardless of the asthma status. The interaction analysis showed that combination of both asthma and obesity had significant effects on IL-5, IL-10, IL-17A, IL-33, TNF-α, and leptin, but the effects were not synergistic or additive (Table 2; Figure 3). This suggests that the presence of asthma and obesity together modifies the inflammatory response differently from each condition alone, with obesity acting as a moderator rather than having a synergistic or additive impact on the cytokine levels in the presence of asthma. For instance, IL-5 levels in children of the OO-A group (13.94 pg/ml) were higher than in those of NW-A group (11.91 pg/ml); both are slightly higher than in children in the OO group (11.49 pg/ml); and markedly higher than children of the NW group (4.66 pg/ml); indicating a moderation effect between excess weight and asthma rather than a synergistic or additive interaction (Table 2).

**Table 2:** Effect of asthma, obesity, and their interaction on inflammatory markers in plasma (*n* = 362).

Plasma inflammatorymarker	*p* value		Normal weight and asthma (NW-A) (*n* = 97)	Overweight obesity and asthma (OO-A) (*n* = 99)	Overweight obesity and no asthma (OO) (*n* = 99)	Normal weight and no asthma (NW) (*n* = 67)
IL-2	O A O-A	0.164 0.005 0.293	2.47 (0.02, 8.84)	2.68 (0.02, 8.71)	1.34 (0.02, 7.17)	0.25 (0.02,2.81)
IL-5	O A O-A	<0.001 <0.001 0.001	11.91 (6.36, 28.02)	13.94 (8.45, 26.64)	11.49 (6.63, 23.65)	4.66 (2.64, 13.83)
IL-10	O A O-A	0.002 0.046 0.029	4.3 (2.38, 10.17)	4.12 (1.98, 8.67)	3.59 (1.21, 8.09)	14.4 (1.39, 56.32)
IL-13	O A O-A	0.115 0.021 0.056	15.27 (2.74, 48.89)	9.31 (0.34, 46.14)	10.89 (0.34, 46.99)	5.01 (0.34, 30.04)
IL-17A	O A O-A	0.002 <0.001 0.014	2.86 (0.04, 5.99)	2.29 (0.04, 7.7)	0.61 (0.04, 5.07)	0.04 (0.04, 1.65)
IL-22	O A O-A	0.030 <0.001 0.079	0.36 (0.04, 0.79)	0.34 (0.04, 0.79)	0.22 (0.02, 0.58)	0.02 (0.02, 0.49)
IL-33	O A O-A	<0.001 <0.001 <0.001	12.93 (4.23, 39.32)	16.92 (2.38, 34.12)	11.9 (5.18, 30.59)	2.1 (0.08, 9.59)
IFN-γ	O A O-A	0.321 0.114–0.204	5.42 (2.13, 12.59)	2.45 (0.1, 8.65)	2.59 (0.1, 7.95)	2.57 (0.1, 34.37)
TNF-α	O A O-A	0.001 <0.001 0.029	8.79 (5.96, 15.24)	10.62 (6.76, 14.01)	8.25 (5.75, 12.98)	6.46 (4.47, 9.5)
Leptin	O A O-A	<0.001 <0.001 <0.001	2,258.26 (960.2, 5,894.8)	8,209.23 (5,684.9, 10,466.8)	9,152.94 (6,690.5, 12,955.6)	1,025.91 (406.7, 1,688.2)

Plasma inflammatory markers are expressed as pg/ml. Cytokines are presented as Median (IQR). All markers were log-transformed in the regression analysis. *p* values are given for the main effects and the interactions:

O, obesity vs. normal weight; A, asthma vs. no asthma; O-A, obesity and asthma interaction. All the *p* values were adjusted for age, gender, and any steroid medication intake.

IFN-γ, interferon gamma; IL, interleukin; TNF-α, tumor necrosis factor-alpha.

### 3.3 Lung function, FeNO, and LCI

Spirometry results indicated that children with asthma had reduced forced expiratory volume in one second (FEV1, *p* = 0.001), FEV1/forced vital capacity (FVC) ratio (*p* < 0.001), and forced expiratory flow midexpiratory phase (FEF25%–75%, *p* < 0.001) compared to children without asthma (Table 3). Children with overweight or obesity had increased values for FVC (*p* = 0.007), compared to those with normal weight. However, no obesity-asthma interactions were observed for any of the spirometry parameters.

**Table 3:** Effect of asthma, obesity, and their interaction on pulmonary function indices.

Variable	*p* values		Normal weight and asthma (NW-A)	Overweight/obesity and asthma (OO-A)	Overweight obesity and no asthma (OO)	Normal weight and no asthma (NW)
			Mean (SD)	Mean (SD)	Mean (SD)	Mean (SD)
FVC	O A O-A	0.007 0.822 0.574	94.4 (13.2)	98.5 (13.5)	100.2 (12.5)	94.3 (12.2)
FEV1	O A O-A	0.069 0.001 0.682	86.7 (15.1)	89.5 (16.8)	98.9 (12.2)	94.7 (12.4)
FEV1/FVC	O A O-A	0.180 <0.001 0.679	80.1 (9.5)	80.0 (8.9)	86.2 (6.4)	88.0 (5.1)
FEF 25%–75%	O A O-A	0.317 <0.001 0.746	68.1 (27.2)	70.7 (25.6)	91.6 (20.0)	87.9 (21.4)
RV	O A O-A	<0.001 0.297 0.045	113.9 (29.5)	101.8 (30.7)	82.5 (25.4)	108.0 (25.5)
TLC	O A O-A	0.941 0.134 0.837	96.4 (11.0)	95.8 (11.2)	93.4 (11.2)	93.0 (11.1)
RV/TLC	O A O-A	<0.001 0.822 0.005	28.5 (6.4)	25.3 (7.5)	21.1 (5.9)	28.6 (5.6)
ERV	O A O-A	0.118 0.389 0.047	110.3 (31.6)	102.8 (34.8)	113.3 (44.6)	105.8 (27.5)
FRCpleth	O A O-A	0.007 0.141 0.913	112.2 (19.7)	102.1 (18.8)	97.0 (19.7)	107.4 (18.7)
IC	O A O-A	0.004 0.611 0.652	81.8 (18.7)	90.4 (20.4)	91.9 (23.1)	80.0 (15.5)
LCI	O A O-A	0.770 0.013 0.870	7.2 (1.3)	7.3 (1.8)	6.7 (0.8)	6.6 (0.6)
FeNO (ppb)^a^	O A O-A	0.052 <0.001 0.584	43.9 (19.4,73.5)	34.4 (16.3,80.0)	15.4 (10.6,27.0)	17.9 (11.8,29.3)

Pulmonary function tests are reported as percent predicted values other than FEV1/FVC and RV/TLC ratios. *p* values are given for the main effects and the interaction between obesity and asthma, adjusted for age, gender, and any steroid medication intake.

A, asthma vs. no asthma; O, obese vs. nonobese; O-A, obesity-by-asthma interaction.

FVC, forced vital capacity; FEV1, forced expiratory volume in one second; FEF, forced expiratory flow midexpiratory phase; RV, residual volume; TLC, total lung capacity; ERV, expiratory residual volume; FRCpleth, functional residual capacity; IC, inspiratory capacity; LCI, lung clearance index; FeNO, fractional exhaled nitric oxide.

^a^ Median (IQR).

Body plethysmography results indicated that children with overweight/obesity had lower resting lung volumes, with lower residual volume (RV, *p* < 0.001) and RV/total lung capacity (TLC) ratio (*p* < 0.001), and higher inspiratory capacity (IC, *p* = 0.004), than normal weight children (Table 3). Among all the parameters measured, only RV was influenced by a significant interaction between overweight/obesity and asthma. As expected, children with asthma had increased fractional exhaled nitric oxide (FeNO, *p* < 0.001) and lung clearance index (LCI, *p* = 0.013) compared to their counterparts without asthma. However, obesity did not influence FeNO or LCI significantly, and there was no interaction effect of obesity and asthma on these parameters (Table 3).

## 4 Discussion

In this study we investigated the relationship between asthma and obesity in children, particularly focusing on their role in modulating inflammatory markers in children. Our findings indicate that several cytokines, including IL-5, IL-17A, IL-22, IL-33, TNF-α, leptin, and IL-10 were independently influenced by asthma and obesity. However, we observed that the interaction between asthma and obesity does not appear to have a synergistic or additive effect on the inflammatory response, but rather a moderating influence.

The children with asthma had an inflammatory profile that was characterized by increased levels of several pro-inflammatory cytokines, including IL-2, IL-5, IL-13, IL-17A, IL-22, IL-33, TNF-α, and reduced level of anti-inflammatory cytokine, IL-10. These findings are consistent with previous studies that have highlighted the central role of Th2 cytokines (e.g., IL-5, IL-13) and Th17-related cytokines (e.g., IL-17A, IL-22) in the pathogenesis of asthma, leading to eosinophilic and neutrophilic inflammation in the airway, and consequently promoting airway hyperresponsiveness and remodeling (22–28). TNF-α promotes the recruitment of innate immune cells, including eosinophils and neutrophils, to the airway during asthma exacerbation (29). Increased plasma levels of TNF-α in asthma have been reported previously (30). In line with that, elevated TNF-α in our study group of children with asthma further suggests persistent airway inflammation.

In children with overweight/obesity, the levels of pro-inflammatory cytokines, particularly IL-5, IL-17A, IL-33, TNF-α, and leptin, were also elevated, which is consistent with previous findings, indicating chronic low-grade inflammation associated with obesity (31–33). Obesity-associated cytokine, leptin, is known to promote pro-inflammatory cytokine release from immune cells, including macrophages and *T*-cells (34, 35).

Increased levels of pro-inflammatory cytokines in obesity-associated asthma compared to normal weight asthma have been reported previously. A study by Maffeis et al. showed that obesity-associated asthma in children is associated with increased serum levels of pro-inflammatory markers including IL-33 and TGF-β1 when compared to children with asthma and normal weight (36). Another recent study indicated that adults with obesity and asthma show slightly increased level of IL-17F and IL-13 compared to those with normal weight and asthma (37). In our study, we also noted a modest increase in the levels of cytokines including IL-2, IL-5, IL-33, and TNF-α. However, we did not see evidence of a synergistic or additive effect of asthma and obesity to exacerbate the inflammation. Instead, the interaction analysis indicated that the presence of obesity in children with asthma primarily led to a modulation of the inflammatory response, as seen with IL-5 levels in children of concurrent asthma and obesity being higher than in those with asthma only, and overweight/obesity only groups but not substantially exceeding the sum of effects seen in either condition alone. Similarly, IL-10 showed consistent decrease in asthma only, and obesity only groups, however, no further significant decrease was observed in children with concurrent obesity and asthma.

To our knowledge, only a few studies have assessed the possible interactions between asthma and obesity on inflammatory cytokines. Sutherland et al. investigated the possible pathological interaction between obesity and asthma in enhancing systemic and airway inflammation in adults (17). They found no obesity-asthma interactions involved in altering the inflammatory markers in the plasma and sputum of adults with obesity and asthma (17). Similarly, Vezir et al., observed no interaction effect of obesity and asthma on serum inflammatory biomarkers such as C-reactive protein, C3, C4, leptin, resistin, periostin, YKL-40, Type 1, and Type 2 cytokines, although they did find a notable interaction with adiponectin (38). Whereas Rastogi et al. found interaction effect between obesity and asthma on the serum cytokines TNF-α, MCP-1, and IP-10 in adolescents with asthma (39). However, no synergistic or additive effect of asthma and obesity interaction on inflammation was observed in these studies.

The observed lack of synergy in above studies as well as our study could be due to the overlap of inflammatory pathways that are activated in both obesity and asthma. Obesity is associated with systemic low-grade inflammation with elevated levels of pro-inflammatory cytokines such as IL-6 and TNF-α (40). Asthma is associated with airway inflammation, with elevated Type 2 and Type 17 mediators (41). It is possible that the inflammatory signals from these two conditions converge on shared pathways, leading to a ceiling effect rather than an additive or synergistic increase.

In our study, we observed that levels of certain cytokines do not directly correlate with peripheral blood cell counts, particularly in the case of overweight/obese asthma group. For example, IL-5 levels were elevated in the overweight/obese asthma group compared to normal-weight asthma group, however there was no corresponding increase in blood eosinophil count. This discrepancy may be due to altered immune regulation associated with obesity. IL-5 is a key cytokine that promotes eosinophil differentiation and survival in the lung (22). However, in the context of obesity, there might be other factors such as adipokines or systemic inflammation, might be regulating the recruitment and activation of eosinophils. These factors can explain the paradox of high IL-5 levels despite lower eosinophil count in the overweight/obese and asthma group as compared to normal-weight and asthma group. Alternatively, there might be differences in tissue distribution of eosinophils between the groups. Further studies, particularly using lung tissue samples are necessary to understand whether overweight/obesity is associated with increased IL-5 production but reduced eosinophil recruitment and activation in the lungs, compared to children with normal weight and asthma.

Additionally, we observed increased neutrophil counts in overweight/obesity and asthma group compared to normal weight and asthma group. However, we did not see corresponding increase in IL-17A levels in overweight/obesity and asthma group. IL-17A is associated with neutrophil recruitment and activation in the lung of asthma (42). The lack of correlation in our study indicates that IL-17A might not be involved in activating neutrophils in children with overweight/obesity. Instead, there might be an alternate pathway that might be playing role in recruiting and activating neutrophils in the context of overweight/obesity and asthma.

The changes observed in IL-10 levels in our study provides valuable insights into potential regulatory mechanisms. IL-10 is a key anti-inflammatory cytokine that modulates immune responses. The reduced levels of IL-10 in both asthma and obesity indicate the common mechanistic link between these both conditions. Reduced IL-10 expression during viral infection in the early life is associated with asthma development by 6 years of age, indicating an important role of IL-10 in asthma development (5). Reduced levels of IL-10 in our overweight/obesity alone group indicate that obesity could act as a contributing factor for asthma development through suppression IL-10 levels.

A major strength of our study is that it was well-designed with appropriate control groups for both asthma and obesity, helping us to assess the individual and combined effects of both conditions on inflammatory profile in children. Another aspect of our study is that it examined the cytokines levels in children with mild and moderate asthma of a well-controlled status; this provides an important information on the inflammatory background of asthma and obesity in the resting state. In contrast, most other studies included severe children with severe asthma or during acute asthma exacerbation. One limitation of our study is its cross-sectional design, which does not allow us to assess the causality. Children might have been asthmatic from their early childhood and developed overweight/obesity at a later age. Nevertheless, we were able to assess the interaction of asthma and obesity on inflammatory markers. Second, the study focused on a limited set of cytokines and adipokines, and future studies should include a broader range of immune markers to provide a more comprehensive picture of immune dysregulation in obese children with asthma. Our study measured the levels of inflammatory markers in the plasma; however, the cell/tissue origin of these markers is unknown. Future studies measuring inflammatory cytokines levels in the airway using bronchoalveolar lavage fluid or sputum samples to measure the markers of airway inflammation including eosinophils, neutrophils, specific IgEs, and cytokines in the sputum might be useful to understand the impact of obesity on airway inflammation. In addition, further studies are needed to confirm our findings in different ethnic and geographic populations.

In conclusion, our findings indicate that several cytokines were independently influenced by asthma and obesity. However, we observed that the interaction between asthma and obesity does not appear to have a synergistic or additive effect on the inflammatory response, but rather a moderating influence. These interactions challenge the previously held view that obesity amplifies the inflammatory response in asthma. However, as mentioned earlier, this observation is limited to the fact that our study was performed in children with a controlled status asthma outside acute exacerbation. Nevertheless, our findings highlight the need to consider the independent and combined contributions of asthma and obesity in managing inflammation in pediatric populations. Since children with concurrent asthma and obesity require increased doses of corticosteroids, it would be interesting to investigate whether these children show a reduced response to steroid therapy compared to their normal weight counterparts.

In light of these findings, further research is needed to better establish the link between obesity and asthma. This will help to adapt adequate therapeutic strategies such as steroid therapy or biologics to effectively treat children with concurrent asthma and overweight/obesity.

## Data Availability

The raw data supporting the conclusions of this article will be made available by the authors, without undue reservation.
